# Interactions between the rabies virus and nicotinic acetylcholine receptors: A potential role in rabies virus induced behavior modifications

**DOI:** 10.1016/j.heliyon.2022.e10434

**Published:** 2022-08-28

**Authors:** Marianne Lian, Karsten Hueffer, Maegan M. Weltzin

**Affiliations:** a University of Alaska Fairbanks, Department of Veterinary Medicine, 2141 Koyukuk Drive, Fairbanks, AK, 99775, USA; b Inland Norway University of Applied Sciences, Department of Forestry and Wildlife Management, Koppang, NO-2480, Norway; c University of Alaska Fairbanks, Department of Chemistry and Biochemistry, 1930 Yukon Dr. Fairbanks, AK, 99775, USA

**Keywords:** Rabies virus, Rabies virus glycoprotein, Nicotinic acetylcholine receptor, Behavior, nAChR

## Abstract

Rabies causes approximately 60,000 casualties annually and has a case fatality rate approaching 100% once clinical signs occur. The glycoprotein on the surface of the virion is important for the host immune response and facilitates interaction of the virion with host cell receptors. Nicotinic acetylcholine receptors were the first receptors identified as a molecular target for the rabies virus. Additional targets, including neural cell adhesion molecule, p75 neurotrophin receptor, metabotropic glutamate receptor subtype 2, and integrin β1, have been added to the list, all of which can mediate viral entry into the cell. Multiple receptors and different subtypes of nicotinic acetylcholine receptors result in a complex picture of virus-receptor interactions. In addition, some data suggest that the rabies virus glycoprotein inhibits cell signaling events mediated by various nicotinic receptor subtypes that have been implicated in altering behavior in unaffected animals. This review focuses on interactions between the rabies virus glycoprotein and nicotinic receptors and proposes possible functional consequences, including behavioral modifications and therapeutic approaches for future research.

## Highlights

• Rabies virus glycoprotein ectodomain binds to nAChRs.
• Glycoprotein and nAChR-selective α-neurotoxins share regions of sequence homology.
• Peptide of the rabies virus glycoprotein modifies behavior in mice.
• Nicotine and genetic deletion of α7 nAChRs modulates pathological aggression.
• We hypothesize rabies virus glycoprotein-nAChR interactions modulate behavior.

## Graphical abstract

**Figure:**
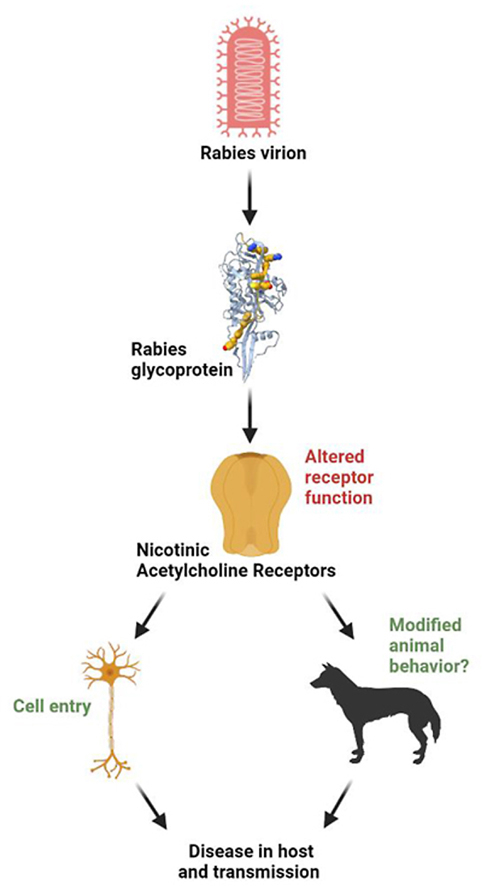

## 1 Rabies background and pathogenesis

The rabies virus (RABV) infects and kills between 50,000 and 70,000 humans each year; mostly in developing countries [1]. As most victims are children, the global impact of >3.7 million Disability Adjusted Life Years better conveys the real burden of dog-mediated rabies due to premature death and adverse events following sub-optimal human rabies vaccinations and treatments [1, 2]. Although rabies has been studied for over 150 years, we still lack a thorough understanding of rabies pathogenesis at the molecular level and thus satisfactory treatment options [3, 4].

The RABV belongs to the Lyssavirus genus of the *Rhabdoviridae* family. The virus is enveloped and has a single-stranded, negative-sense RNA genome, containing five genes which code for nucleoprotein (N), phosphoprotein (P), matrix protein (M), glycoprotein (G), and a viral RNA polymerase (L) (see reviews Fooks et al. 2017 and Jackson 2020 for more detailed information). The RABV glycoprotein (RGP), which interacts with cellular targets, is arranged on the surface of virus particles forming trimers, containing cytoplasmic, transmembrane, and ectodomains [5, 6]. Specifically, the ectodomain homotrimers of the RGP are responsible for interacting with host cell receptors, including the nicotinic acetylcholine receptor (nAChR) [7], neural cell adhesion molecule (NCAM) [8], p75 neurotrophin receptor (p75NTR) [9], metabotropic glutamate receptor subtype 2 (mGluR 2) [10], and integrin β1 [11]. These receptor-RGP interactions initiate viral endocytosis to enter the cell [4, 12].

The RABV shows strong neurotropism, however, the histopathological findings seen after lethal RABV infection are often subtle and do not match the severity of the clinical signs [13]. RABV infection of neurons causes degenerative changes in dendrites and axons as well as oxidative stress caused by mitochondrial dysfunction [14, 15]. Further, neuronal blebbing, apoptosis, and altered neurotransmitter function have been observed after RABV infection [16, 17]. Apoptosis can be detected during late stages of the infection [18]. Nevertheless, how viral infection and neurotropism translate to disease is still unclear [3]. RABV infection in humans can lead to characteristic behavior changes such as hyperactivity, confusion, drowsiness, and agitation [4]. The molecular mechanisms leading to these extensive behavior changes, including aggression, hyper sociability, hydrophobia, and hypermobility, are uncertain [19], although newer reports have started to unveil some possible mechanisms [3, 4, 20].

Rabies has a case fatality rate approaching 100% once clinical signs occur owing to a lack of satisfactory treatment options once the virus has entered the central nervous system (CNS). However, the development of disease after exposure to the RABV can reliably be prevented with very effective post exposure prophylaxis. Limited access to post exposure prophylaxis is a major barrier to eliminating human disease and resulting fatalities. An improved understanding of the cellular mechanisms altered by RABV infection will likely lead to identification of new therapeutic targets and result in better treatment strategies as well as an amended general comprehension of rabies pathogenesis. Since several characteristics of rabies pathogenesis and ecology fall outside the norms of many viruses, an enhanced understanding of rabies pathogenesis will likely further our understanding of virus-host interactions. In this review, we will discuss the interactions between the RABV and host receptors with an emphasis on nAChRs, compare the behavioral modulation asserted by nAChR and RABV both individually and their possible interaction, as well as propose possible mechanisms and examples of the therapeutic implication of these interactions. These proposed mechanisms are based on a limited number of studies from different fields such as cancer biology and rabies pathogenesis, and open possible avenues for further research to better understand the pathogenesis of this important neglected infectious disease.

## 2 Cell surface receptors targeted by the RABV

The RGP is the only external protein on the rabies virus particle, making the RGP the key factor mediating virus entry into cells and the major target for neutralizing antibodies [5]. The function of the RGP as an antigen and the mechanism it utilizes to interact with cell surface receptors have been described [12, 21]. So far, four different receptors, in addition to nAChRs, have been identified as targets for RGP: NCAM, p75NTR, mGluR 2, and integrin β1.

The NCAM is a glycoprotein concentrated in synaptic regions and neuromuscular junctions (NMJs) [12]. There are three major isoforms of NCAM, -120, -140, and -180, which are generated by alternative mRNA splicing from a single gene [22, 23]. NCAM-140 and -180 are attached to their plasma membranes via transmembrane domains [24]. NCAM-120 has a glycosyl-phosphatidylinositol anchor that serves as the attachment point to the cell membrane. All three NCAM isoforms are targets for RABV [8, 25].

*In vitro* studies found that incubation with RABV decreased surface expression of NCAM and that treatment of susceptible cells with heparan sulfate, a ligand for NCAM, or with NCAM antibodies, significantly reduced RABV infection [8]. Pre-incubation of RABV inoculum with soluble NCAM protein, functioning as a receptor decoy, drastically neutralized the capacity of RABV to infect susceptible cells*. In vivo* studies using NCAM deficient mice showed a delay in rabies mortality as well as drastically restricted brain invasion by RABV [8]. These studies support the role of NCAM for RABV infection. Although, neuronal RABV infection in the absence of NCAM receptors can still occur [25], suggesting involvement of other receptors and mechanisms in neuronal RABV infection.

The p75NTR is also targeted by RABV. *In vitro* experiments showed that cells expressing p75NTR bind RGP, and that β-NGF, a p75NTR ligand, inhibits RABV infection [9]. p75NTR is mainly expressed during development, but in adult individuals it has important functions in hippocampal synapse modification [26, 27], the regulation of neurogenesis [28], and inhibition of axon regeneration after peripheral nerve injury [29]. Even though the p75NTR is not essential for RABV infection [30], it may be important for facilitating RABV transport to the CNS [31]. Live-cell imaging of sensory dorsal root ganglion neurons directly demonstrated that p75NTR co-internalizes with RABV and, subsequently, undergoes retrograde axonal transport [31]. The p75NTR binding site has been proposed to be located outside of the known antigenic site but within residues 318–352 of the RGP, since an anti-RGP antibody did not neutralize the p75NTR site [32].

The mGluR 2 is another cellular target for the RGP that facilitates virus entry into neuronal cells [10]. The mGluR2 belongs to a class of seven transmembrane domain receptors. It is abundant in the CNS and rarely expressed in other tissues [33]. Using flow cytometry, RABV infection decreases cell surface expression of mGluR2 [10,21]. Providing further support that mGluR2 are a RABV target, mGluR2 antibodies reduce RABV infection *in vitro* [10]*.* Additionally, mGluR2 ectodomain soluble protein neutralizes the infectivity of RABV cell-adapted strains *in vitro* and *in vivo* in a dose-dependent manner.

Most recently, integrin β1 was identified as a possible host-receptor for RABV and shown to be involved in peripheral infection [11]. Integrin β1 is abundantly expressed in skeletal muscle but is rare in the cerebral cortex [11]. RABV and integrin β1 directly interact via a coimmunoprecipitation, and co-internalize into cells via a clathrin-dependent endocytosis after RABV infection. Murine *in vivo* studies revealed by immunohistofluorescence that integrin β1 and RABV co-colocalized in muscle, but not in cerebral cortical neurons.

## 3 Neuromuscular nAChR

The first receptor identified as a molecular target for the RGP was the muscle subtype of nAChRs found at post-synaptic sites in the NMJ [7, 12]. Both the neuromuscular and the neuronal nAChRs are pentameric, cation-conducting channels which respond to the endogenous neurotransmitter acetylcholine and are involved in signal transduction. In mammals, there are 16 subunits, which are designated α1 – α7, α9, α10, β1 – β4, δ, ε, and γ based on sequence homology. The α2 – α7, α9 – α10, and β2 – β4 subunits are expressed by neurons and are referred to as the neuronal nAChR subunits [34, 35]. The mammalian muscle nAChR localized to the NMJ is composed of (α1)_2_β1δγ subunits in developing muscle, with the ε subunit substituted for the γ subunit in mature muscle [36].

Seminal work by Lentz and colleagues in the 1980s identified the NMJ nAChR to be important for RABV infection initially in muscle cells prior to infection of the CNS. Pretreatment of α-bungarotoxin and d-tubocurarine, ligands with high nAChR specificity, reduced attachment and infection of two strains of RABV in chick embryo myotubes with robust nAChR expression [37]. Further, gold-labeled RABV antigen and particles were found at the junctional folds of the NMJ, which highly express nAChRs [38]. In nerve-muscle coculture, the rabies virus preferentially localized to the NMJ which expressed α-bungarotoxin labeled nAChRs, and within endosomes at nerve terminals [39].

At the NMJ, the α1 subunit of the muscle subtype of nAChR is abundant and has been shown to facilitate RABV peripheral infection *in vivo* [37]. Interestingly, an interaction between integrin β1 and the α1 nAChR subunit was detected in a coimmunoprecipitation assay with transfected HEK293 cells, indicating that the integrin β1 role in RABV peripheral infection involves the muscle nAChR subtype [11].

## 4 Neuronal nAChR

Dorsal root ganglion (DRG) neurons are believed to be a crucial bridge allowing RABV to pass from the periphery into the CNS. Adult mouse DRG cells treated with non-nAChR subtype-selective antagonists mecamylamine and d-tubocurarine reduced the percentage of RABV infected neurons [40]. Interestingly, the DRG expresses a variety of neuronal nAChR subtypes, including α7, α3β4∗, and α6β4∗ (where ∗ indicates other subunits may be present) [41, 42]. These results suggest that neuronal nAChRs are possible targets for RABV.

Neuronal nAChRs located in the CNS have important physiological roles including mediating cholinergic excitatory neurotransmission, neurotransmitter release, and downstream intracellular pathways [43]. The nAChR subunit composition determines the pharmacological and biophysical properties of these receptors, and individual receptor subtypes are associated with a wide variety of biological processes, including regulation of animal behavior [34, 35], that are altered during RABV infection.

During rabies, the host has altered locomotor behavior, including increased ranging distance, hyperactivity, and ataxia [44, 45], processes which are also modulated by nAChRs. For example, the α4β2 and the α7 subtypes, the most common nAChRs in the CNS, are linked to hyperactivity, aggression, and anxiety [46, 47, 48, 49]. Locomotor behavior involves the nigrostriatal and mesolimbic dopaminergic systems which express the α6β2β3, α4α5β2, and α4α6β2β3 nAChR subtypes [50, 51, 52, 53, 54, 55, 56]. The α3β2 and α3β4 subtypes are expressed in the brainstem [49, 57, 58], which has integrative functions, including controlling the cardiovascular system, respiration, awareness, consciousness, and muscle contraction [59], processes also modified with RABV infection. These correlations, while not experimentally confirmed suggest a possible role for these neuronal receptors in rabies pathogenesis.

The function of neuronal nAChR subtypes in RABV infection is currently not well understood, although progress is being made [12, 21, 60]. Rat hippocampal cell culture neurons have been shown to be highly susceptible to infection by RABV [61]. Entry of the virus appears to predominantly occur in regions of the cell that highly express nAChRs, including the cell body, dendrites, and in synapses, with very little viral antigen present in axons.

A 2017 study by Hueffer and colleagues, using a combination of *in vivo* and *in vitro* methods, found that administration of the neurotoxin-like region of the RGP (see section below) led to behavior modifications, possibly through inhibition of the α4β2 nAChR subtype in *C. elegans* and mice [20]. The neurotoxin-like region of RGP inhibited the frequency of nAChR-meditated pharyngeal pumping in *C. elegans* and induced hyperactivity (a rabies-associated behavior) after intraventricular administration into the CNS of mice. Additionally, the α4β2 nAChR subtype was inhibited by both the neurotoxin-like region of RGP and the ectodomain of RGP in functional assays using a two-electrode voltage clamp electrophysiology [20]. RABV and host nAChR interactions are traditionally viewed to mediate mechanisms of viral cell entry and immune recognition [4]. However, the study by Hueffer et al. [20] suggests that this could be expanded to include functional modification of receptor signaling and even specific behavior modifications of the infected hosts.

## 5 RGP binds to nAChRs via a neurotoxin-like domain

The RGP contains a neurotoxin-like region which shows a significant sequence homology with snake α-neurotoxins that function as potent nAChR subtype selective antagonists [62, 63]. Several lines of evidence have identified the α1 nAChR subunit to contain the RGP binding site, which overlaps with the α-bungarotoxin site. nAChR α1 subunit monoclonal antibody prevented the attachment of radio-labeled RABV to cultured muscles cells with a high density of nAChRs [38]. RABV binding to the muscle-type nAChR was inhibited by nAChR antagonists, up to 50% by α-bungarotoxin and up to 30% by (+)-tubocurarine, but binding was not affected by the muscarinic acetylcholine receptor antagonist atropine [64]. Later, the RABV was confirmed to bind to the *Torpedo californica* electric organ α subunit, which is similar to the human α1 subunit, by a competitive mechanism, as α-bungarotoxin was able to compete for binding [65].

The RGP neurotoxin-like domain is located between residues 175–203 of the mature RGP and has homologies to loop II (the central loop) of the α-neurotoxins and α-conotoxins (Table 1) [66-68]. A 2021 review contains detailed structural information of venom-derived neurotoxins and the ability of these proteins to target nAChRs [69]. Therefore, we have focused our discussion to possible parallels that can be surmised from α-neurotoxin loop II and interactions with nAChRs, and the similarities to the RGP.

**Table 1:** Sequence comparison between RVG and loop II of α-bungarotoxin and α-cobratoxin. Bolded residues are conserved among RGP and at least one α-neurotoxin. The underlined residues are those important for mediating nAChR interactions. Snake toxin residues absent in RGP are represented with a dash space holder to facilitate sequence alignments.

Virus/Toxin	Sequence																														
RGP	**Y**	**T**	-	I	**W**	M	P	E	N	P	R	L	G	T	S	**C**	**D**	I	**F**	T	N	**S**	**R**	**G**	**K**	**R**	A	S	K	**G**	203
α-Bungarotoxin	**Y**	R	K	M	**W**	-	-	-	-	-	-	-	-	-	-	**C**	**D**	A	**F**	C	S	**S**	**R**	**G**	**K**	V	V	E	L	**G**	43
α-Cobratoxin	**Y**	**T**	K	T	**W**	-	-	-	-	-	-	-	-	-	-	**C**	**D**	A	**F**	C	S	I	**R**	**G**	**K**	**R**	V	D	L	**G**	40

Loop II of α-bungarotoxin and α-cobratoxin have approximately 50% sequence homology to the RGP neurotoxin-like domain (Table 1). Circular dichroism spectroscopy identified that a 29 amino acid residue peptide of the neurotoxin-like domain of the RGP and an analogous king cobra loop II peptide were conformationally similar and were composed mostly of beta sheet structure [70]. This study showed that a peptide of the RGP neurotoxin domain is structurally similar to loop II of α-neurotoxins. Using a Asn194-Ser195-Arg196-Gly197 tetrapeptide, early molecular modeling studies showed that these RGP residues form an essential part of the binding site and that side chains Asn and Arg demonstrate molecular mimicry to the structure of ACh [71].

X-ray crystallography and cryo-EM structures have revealed great detail into the interactions of α-neurotoxin loop II with nAChRs [72, 73, 74, 75]. In the *Torpedo* receptor, α-bungarotoxin loop II reaches under loop C, penetrating deeply into the ACh binding pocket [74, 75]. The α-bungarotoxin Arg 36 guanidinium group is located far within the ACh binding pocket and contacts the principal face α subunit residues Tyr93, Tyr190, and Tyr198 and forms a cation-π sandwich with αTyr198. α-bungarotoxin loop II Phe 32 interacts with residues α1Trp149 and γTrp55 or δTrp57, which are important for ACh binding, while Val 40 makes important contacts with Tyr189 [74]. A cryo-EM structure of the α7 nAChR showed that the α-bungarotoxin Arg36 and Phe32 form a cation-π stack and aligns with Tyr187 in an edge-to-face orientation [75].

Several loop II residues of α-neurotoxins are important for ligand affinity and subtype specificity [68, 72, 76, 77, 78]. Mutating α-bungarotoxin residues Phe 32, Arg 36, and Gly 43 reduced binding affinity for *Torpedo* nAChR, while mutating similar RGP peptide residues showed analogous affects [68]. Similarly, α-cobratoxin residues Trp 25, Asp 27, Phe 29, Arg 33, and Arg36 are implicated in the binding of muscle and α7 nAChR subtypes [72]. Loop II residue Lys 23 is important for binding to the muscle subtype, while Ala 28, Cys26-Cys30, and Lys 35 are important for binding to the α7 nAChR subtype [72]. These findings show that functional similarities possibly exist between some α-neurotoxins and the RGP, but it remains to be determined if RGP selectively targets subtypes of neuronal nAChRs. The structural and sequence resemblances of α-neurotoxins and RGP opens the door to better understand the role of RGP in the pathogenesis of rabies beyond cell entry, as well as improving therapeutic approaches for rabies and other diseases associated with nAChR dysfunction, such as Alzheimer's disease, Parkinson's disease and schizophrenia [79, 80].

## 6 RGP structure

**Figure 1:**
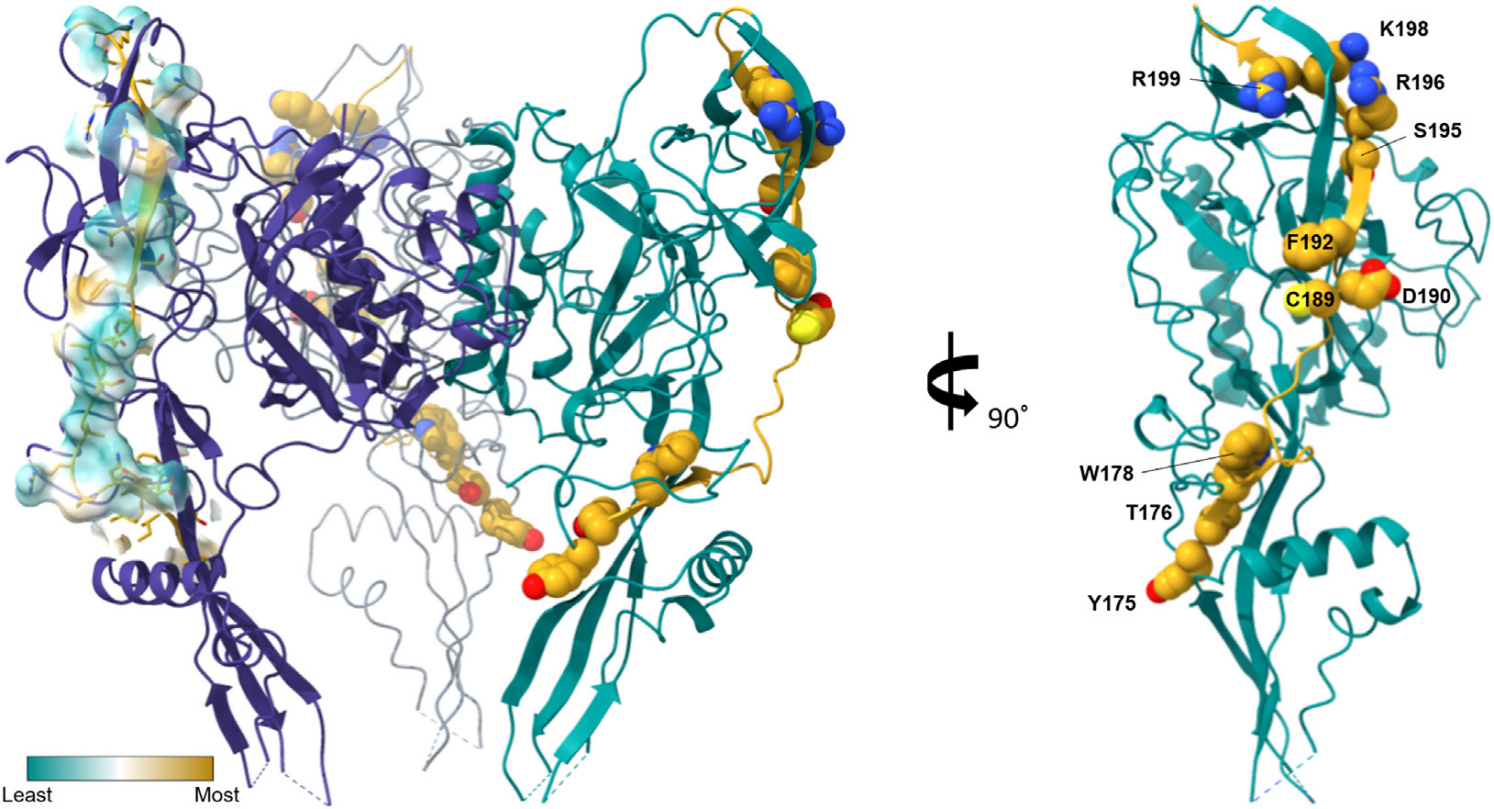
**Rabies glycoprotein neurotoxin-like loop residues are accessible to solvent in the solved prefusion RGP structure.** The yellow region represents the neurotoxin-like domain (residues 175–203) found in the PHD (domain III). Space filled and labeled residues correspond to those important for α-neurotoxin interactions with nAChRs. In the trimer model, the hydrophobicity of the neurotoxin-like domain of the left protomer is displayed demonstrating that the region is largely hydrophilic (cyan), with several key exposed residues being hydrophobic (yellow). A single protomer rotated 90° is shown on the right. Model was built using UCSF ChimeraX (version: 1.40.93 (2020-06-03)) and the solved cryo-EM structure of RGP timer (PDB 7U9G) [6].

Homology modeling with the vesicular stomatitis virus (VSV) glycoprotein predicted that the RGP neurotoxin-like domain is exposed on the protein surface [81]. In 2020 x-ray crystallography studies, the structure of the RGP was solved [5, 82] and largely confirmed the predictions that were based on the VSV glycoprotein structure [83, 84]. In 2022, Callaway et al. published the structure of the RGP trimer bound to a prefusion-specific neutralizing antibody [6]. The glycoprotein ectodomain is divided into four [6, 82] linked domains: a central domain (CD, or domains I and II), a pleckstrin homology domain (PHD, or domain III) and a fusion domain containing two fusion loops (FD, or domain IV). The CD is connected to the transmembrane and cytoplasmic domains of the RGP at the C-terminus, with a central helix that elongates in the postfusion transition (domain II) and a solvent exposed upper half of the RGP (domain I). The presumed binding site of the RGP to nAChRs (see section above) is found in the PHD (domain III) which is also largely solvent exposed in the upper half of the RGP [5, 6] (Figure 1). However, it seems that in the Yang et al. [5] pre-fusion RGP structure, the neurotoxin-like domain does not fold into a loop structure that is typical for the homologous regions in neurotoxins, likely due to the lack of disulfide bonds. In this structure, the RGP neurotoxin-like region forms a β-strand, followed by a random coil, and ending with another β-strand (Figure 1), which is the same motif found in α-neurotoxins. Interestingly, the residues (175–203) that have been found to be critical for α-neurotoxin binding to nAChRs (see Table 1), are highly exposed to solvent, suggesting these residues to be accessible for nAChR interactions (Figure 1). Mapping the lipophilicity potential of this region shows that the region is highly hydrophilic, which makes sense due to its solvent accessibility, thus the residues likely participate in hydrogen and electrostatic interactions. Several residues including Cys 189. Phe 192, Arg 196, and Lys 198 likely interact with target residues via hydrophobic interactions. Residues Tyr 175 and Thr193 may also interact with nAChRs but are less obvious due to the residues positioning. Detailed structure and function studies of the RGP and nAChR interactions are needed to confirm these observations.

## 7 nAChR modulational of behavior

The traditional virus interaction with cell surface receptors as a mechanism of cell entry may not be the only consequence of RGP-nAChR interactions. Here we discuss the possibility that inhibition of nAChRs by the RGP may play a key role in the dramatic behavior changes observed with RABV infection [20].

The neuroanatomical bases and neurological mechanisms for the behavioral changes observed during RABV infection are not well understood. Infection of the limbic system and subsequent dysfunction is suspected to play an important role in host behavior [3]. Two-thirds of humans infected with dog RABV variants present with the classic furious rabies, characterized by fluctuating consciousness, changed mental status, aggression, hydrophobia, and autonomic stimulation signs [19]. The remaining third develop paralytic rabies progressing to coma [19]. The mechanisms determining the form of RABV infection is not known but does not seem to be associated with RGP sequence [85].

Increased movement, aggression, and biting in animal hosts are important for transmission of the RABV to new hosts at a time when the virus is secreted in saliva. This aggressive behavior may be associated with low serotonergic activity in the brain [86, 87]. Accumulation of RABV antigen was found in the midbrain raphe nuclei in experimentally infected skunks, indicating impaired serotonin neurotransmission from the brainstem may contribute to the aggressive behavior [88]. Combining molecular techniques and animal models revealed that the RGP derived neurotoxin-like peptides modify host behavior, such as inducing hyperactivity in mice, possibly by inhibition of neuronal nAChRs [20]. However, this study did not specifically antagonize nAChR subtypes, or other RABV receptors.

nAChRs are known to modulate behaviors including aggression, attention, mood, and impulsivity. Nicotine and other drugs targeting nAChRs can reduce offensive, defensive, and predatory aggression in animal models [89]. Correspondingly, in human laboratory and clinical settings, nicotine may reduce aggressive behavior [90, 91, 92, 93, 94, 95], and the α7 nAChR subtype may be a critical component to modulating this aggressive behavior. The α7 nAChR is necessary for the anti-aggressive or ‘serenic’ effects of systemic administration of nicotine, and an α7 nAChR partial agonist (GTS-21) can reestablish this serenic nicotinic effect [46].

The hippocampal α7 nAChR seems to directly regulate aggression in mice [96]. The loss of α7 nAChR subtype function, either by pharmacological means in mice or by genetic deletion in humans as occurs with the 15q13.3 microdeletion syndrome, increases aggression [96, 97, 98, 99]. What remains to be determined is whether RGP functionally interacts with the neuronal α7 nAChR subtype and can modify animal behaviors including aggression. These rodent and human studies provide evidence that the neuronal α7 nAChR plays a critical role in modulating aggression behaviors and that this behavior can be modified with exogenous ligands. The RGP with its neurotoxin-like region could be one such ligand. Further experimental support is needed to test this hypothesis. These experiments should include specific behavioral assays to test effects on aggression as well as knock out mice to evaluate the role of different subtypes of nicotinic receptors.

## 8 Therapy development based on functional RGP and nAChR interactions

In addition to providing a possible mechanistic explanation for rabies-associated behavioral changes, the functional RABV interaction with nAChR is being used for the development of therapeutic approaches to treat cancer and rabies.

Controlling cancer cell replication by limiting cell division through modification of intracellular signaling pathways provides a promising avenue for the development of cancer treatment. For some lung cancers, upregulation of α7 nAChRs has been observed compared to precancerous cells and is related to smoking [100]. The RGP's affinity to the nAChR has been utilized in the development of possible lung cancer treatments. The oncolytic Newcastle Disease Virus expressing the RGP enhances apoptosis and inhibits migration of lung adenoma cells by regulating α7 nAChR signaling pathways [100]. Similarly, by antagonizing α7 nAChR, the RGP promotes apoptosis in gastric carcinoma cells, demonstrating the potential of RGP for treatment of gastric cancer [101]. These interaction between RGP and α7 nAChR not only provide some evidence of the usefulness of RGP nAChR interaction in developing therapeutic approaches to non-rabies diseases but also further strengthen the proposed role in rabies pathogenesis suggested in this review article.

Interactions between α1 nAChR derived peptides with the RGP were investigated for the purpose of designing potential anti-rabies agents. α1 nAChR peptide sequences from different host species (*bovine*, human, electric fish/*torpedo*) were tested, and both the *bovine* and *torpedo* peptides bound and inhibited the RGP [102]. Encouragingly, α1 nAChR peptides and their analogs may serve as potential leads in developing antiviral agents against RABV infection.

In order to develop better treatment strategies, it is critical to understand the process by which the RABV is able to evade the host immune response and gain access to the CNS. A recombinant trimeric RGP binds to α7 nAChRs expressed on monocyte-derived macrophages [103]. This interaction induced the cholinergic anti-inflammatory pathway, including suppression of macrophages to function as T-cell activators, and may affect macrophage polarization. These findings suggest that RABV could evade the immune system by inducing an anti-inflammatory state in human macrophages through interactions with α7 nAChR.

## 9 Conclusion

The studies reviewed in this manuscript show that rabies virus interacts with multiple host target receptors, including nAChRs. The effects of RGP binding to neuronal nAChRs possibly results in inhibition of receptor function and alterations of associated signaling pathways, and we propose a potential link to RGP modifying animal behavior. These findings suggest the possibility of a complex relationship of RABV and its interaction with host cells through the RGP.

We propose that the RGP can function as a neuronal nAChR antagonist and could induce aggression, and possibly other behaviors associated with rabies. Our suggestion is based on five main conclusions: (**1)** The RABV binds to muscle and neuronal (α4β2) nAChRs via the RGP ectodomain [20, 65, 68, 70, 71, 104]. (**2**) α-neurotoxins are known to bind at the orthosteric binding site on muscle and neuronal (α7) nAChRs [72, 74, 75]. (**3)** RGP and snake α-neurotoxins have relatively high sequence homologies, and α-neurotoxins can compete for binding with RGP on muscle nAChRs [64, 65, 66, 67, 68, 77, 105]. (**4)** Nicotine and genetic deletion of the α7 nAChR can significantly modulate pathological aggression [46, 90, 91, 92, 94, 95, 96, 97, 98, 106]. (**5)** RABV binds to the α7 nAChR, inducing an anti-inflammatory state and altering intracellular signaling in several cell types [100, 103, 107]. Together, these outcomes suggest the possibility that the RGP binds to neuronal nAChRs, to possibly modulate aggressive behavior in host animals, which would aid in the transmission of the virus. These proposed mechanisms currently lack strong experimental support but could provide a fruitful avenue to better understand rabies pathogenesis, and encourages further experimental research on the topic.

The existence of multiple receptors for the rabies virus on host cell membranes complicates interpretations of some *in vitro* and *in vivo* results. Expanding our understanding of virus-receptor interactions beyond cell entry to include alterations in host cell biology and behavioral modifications could lead to fundamental advances in the field of virial pathogenesis and host-pathogen interactions. These findings suggest the need for further studies of the functional aspects of virus-receptor interactions and how changes in receptor function alter host cell biology beyond virus entry. Only with a better understanding of host factors and RABV pathogenetic mechanisms can a safe and effective antiviral therapy be developed.

## Declarations

### Author contribution statement

All authors listed have significantly contributed to the development and the writing of this article.

### Funding statement

Karsten Hueffer was supported by National Institute of General Medical Sciences of the National Institutes of Health; Institutional Development Award (IDeA) [P20GM103395].

### Data availability statement

No data was used for the research described in the article.

### Declaration of interest's statement

The authors declare no conflict of interest.

### Additional information

No additional information is available for this paper.
